# The efficacy of intramuscular electrical stimulation in the management of patients with myofascial pain syndrome: a systematic review

**DOI:** 10.1186/s12998-021-00396-z

**Published:** 2021-09-27

**Authors:** Monavar Hadizadeh, Abbas Rahimi, Mohammad Javaherian, Meysam Velayati, Jan Dommerholt

**Affiliations:** 1 Department of Physiotherapy, School of Rehabilitation, Shahid Beheshti University of Medical Sciences, # Damavand Ave, Zip code:16169-13111 Tehran, Iran; 2 Department of Physiotherapy, School of Rehabilitation, Tehran University of Medical Sciences, Tehran, Iran; 3 Musculoskeletal RadiologistDepartment of Radiology, Akhtar Orthopedic Hospital, Shahid Beheshti University of Medical Sciences, Tehran, Iran; 4 Bethesda Physiocare, Bethesda, MD USA; 5 Myopain Seminars, Bethesda, MD USA; 6 Department of Physical Therapy and Rehabilitation Science, School of Medicine, University of Maryland, Baltimore, MD USA

**Keywords:** Intramuscular electrical stimulation, Myofascial pain syndrome, Trigger point, Dry needling

## Abstract

**Introduction:**

Myofascial pain syndrome (MPS) is one of the most common disorders causing chronic muscle pain. Almost one-third of patients with musculoskeletal complaints meet the MPS criteria. The aim of this study is to evaluate the effectiveness of intramuscular electrical stimulation (IMES) in patients with MPS through a systematic review method.

**Methods:**

PubMed, Scopus, Embase, ProQuest, PEDro, Web of Science, and CINAHL were systematically searched to find out the eligible articles without language limitations from 1990 to December 30, 2020. All relevant randomized controlled trials that compared the effectiveness of IMES with sham-IMES, dry needling, or exercise therapy in patients with MPS were included. Full texts of the selected studies were critically appraised using Revised Cochrane risk-of-bias tool for randomized trials (RoB2).

**Results:**

Six studies (out of 397) had met our inclusion criteria (involving 158 patients) and were entered to the systematic review. Outcome measures examined in these studies included pain, range of motion, pressure pain threshold, biochemical factors, disability, and amount of analgesic use. In the most studies, it has been shown that IMES is more effective than the control group in improving some outcome measurements such as pain.

**Conclusion:**

There is preliminary evidence from a few small trials suggesting the efficacy of IMES for the care of myofascial pain syndrome. The data support the conduct of larger trials investigating the efficacy of IMES.

**Supplementary Information:**

The online version contains supplementary material available at 10.1186/s12998-021-00396-z.

## Introduction

Myofascial pain syndrome (MPS) is one of the most frequent disorders causing chronic muscle pain that is usually overlooked [1]. Almost one-third of patients with musculoskeletal complaints meet the Simons and Travel MPS criteria [2]. Myofascial Pain Syndrome originates from a sensitive zone, referred to as a trigger point (TrP) [3, 4]. A trigger point is a painful point within a muscle contracture or taut band in the muscle belly, which is aggravated by a directly applied force, pressure, contraction, or stretching. A trigger point can cause referred pain to remote areas, limited range of motion (ROM), and reduced functional ability [2, 4–7].

Different physiotherapy interventions have been recommended to manage MPS, such as electrotherapy, manual therapy, exercises, and dry needling (DN) [8–12]. Current articles report evidence with different levels of effectiveness and long-term effects of physiotherapy interventions, including manual therapy, electrotherapy, and dry needling of TrPs. Therefore, it seems that further research is still needed to provide appropriate treatment for TrPs [8, 13]. Based on previous study results, DN positively affects the signs and symptoms of MPS [10]. There is also some evidence that electrical stimulation (ES) can increase blood flow to the muscle [14, 15]. Some researchers have combined DN with ES to achieve more effective treatment outcomes for blood flow, pain severity, and ROM, among others [16, 17].

There is some evidence of the effectiveness of intramuscular electrical stimulation (IMES) applied to various body regions in patients with MPS; however, these studies have much heterogeneity, making it difficult to draw definitive conclusions and apply the results in clinical practice. Therefore, we conducted a systematic review of randomized controlled trials (RCTs) to evaluate the effectiveness of IMES in the management of patients with MPS.

## Methods

### Inclusion/exclusion criteria

#### Type of studies

Any published RCTs reporting the effects of IMES on myofascial pain were included in this systematic review with no language restriction. Studies were considered eligible included patients with MPS based on Simons and Travel MPS criteria [2].

Also, only studies were included with patients with MPS.

#### Type of participants

Patients with MPS in any body region, sex, gender, and age were included.

#### Type of interventions

We include all RCTs applied IMES using DN with all types of wave properties. Anode or cathode use on TrP was not important for study including. All intervention types except IMES by DN, such as DN alone, ES insertion without DN use, or no intervention were considered proper for the control group.

#### Type of outcome measurements

Any quantitative outcome measurements like pain, ROM, functional disability score, etc., were accepted into the current study.

### Search methods

Two researchers (MH & MJ) independently searched seven relevant databases to identify potentially relevant studies, including PubMed, Scopus, Embase, ProQuest, PEDro, Web of Science, and CINAHL from 1990 to December 2020. To identify keywords, the terms myofascial pain, trigger point, and intramuscular electrical stimulation were searched in medical subject heading (MeSH), and their synonyms were included in searching the databases. The searched keywords were ("Intramuscular electrical stimulation" OR "electrical intramuscular stimulation" OR "intramuscular stimulation" OR IMES OR EIMS OR "electrical twitch") AND ("trigger point" OR myofascial OR muscle OR muscular). The authors also searched the included articles' references and consulted the … University of Medical Sciences library to identify other relevant studies.

### Study selection and data extraction

Two researchers (MH & MJ) independently screened the title and abstract of all identified articles. During the next stage, they reviewed the full texts of all potentially relevant studies. Researchers read the articles independently and extracted the data based on a pre-determined datasheet. The extracted data included study design, sample size, type of MPS disorder, age, interventions in experimental and control groups, number and frequency of treatment sessions, location of treatment, wave characteristics, needling method, outcome measures, and study results. We used Google Translate online software to extract data from non-English article [18, 19].

### Risk of bias assessment

We used Revised Cochrane risk-of-bias tool for randomized trials (RoB2) to evaluate the quality of included studies. This tool has five parts that include the Randomization process, Deviations from the intended interventions, Missing outcome data, Measurement of the outcome and Selection of the reported result. The overall bias for each study is based on the bias level obtained in each of these sections [20]. Any disagreements between the two researchers regarding the inclusion and quality assessment processes were resolved by an expert researcher (AR).

### Statistical analysis

In this study, descriptive statistics are presented, including the means and SDs and statistical significance for between-groups comparisons for each outcome at each follow-up time point (“Appendix 1: Table 3”). Because of the small number of included studies and clinical heterogeneity discussed under the limitations section, data could not be pooled and meta-analysis on the results.

## Results

**Fig. 1:**
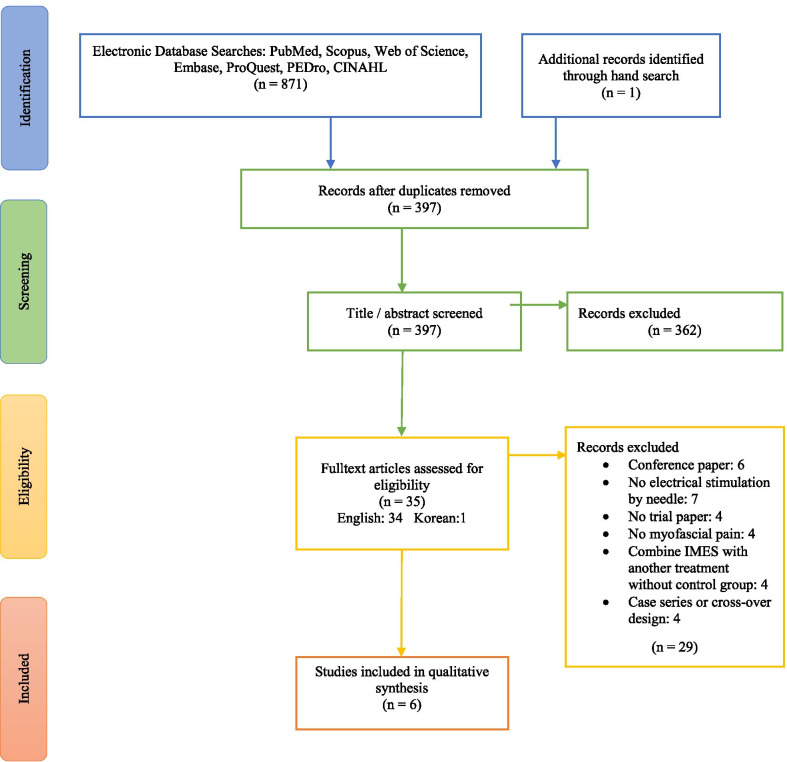
PRISMA flow diagram

After searching the databases and removing duplicate items, 397 potentially relevant titles and abstracts were identified. After screening the title and abstracts, 362 articles were excluded. Thirty-five studies were selected for full-text review. Finally, six studies were included in this systematic review based on the inclusion and exclusion criteria. The most frequent reasons for excluding studies were unrelated titles during the initial review, studies without electrical stimulation application via a needle, conference papers, etc. The details of the excluded studies with justification for exclusion are presented in the “Appendix 2: Table 4”. The process of searching and screening is summarized in Fig. 1.

### Characteristics of included studies

A summary of the methodological characteristics of the included studies and their results are presented in Table 1. Among the selected studies, five studies were in English [21–25], and one study was in Korean [26]. Two studies used patient and assessor blinding [22, 24], and two studies only used assessor blinding [21, 23]. Two studies did not include any blinding [25, 26].

**Table 1:** Summary of the study design, participants characteristics, outcome measurements, assessment time and summary of results of included studies

First author (year)	Type of disorder	Sample size (F/M) (n)	Age (Mean ± SD)	Exp. group	Cont. group	Outcome measurement	Time of assessment	Main results
Byeon [26] (2003)	UT MPS	20 (8/12) Exp.: 10 Cont.: 10	Total participants: 50.7 ± 10.1	IMES	DN	VAS MPQ Neck ROM (lateral flex.)	Before Three days One week Two weeks	No significant difference of all outcome measurements between groups in all assessment times
Sumen [21] (2015)	MPS	30 (22/8) Exp.: 15 Cont.: 15	Total Participants: 38.6*	SIMES + Stretching exercise	Home-based stretching exercise twice daily (10 repetition)	VAS PPT Neck ROM (opposite side lateral Flex.) NDI	Before After One month	Significantly VAS decrease & PPT increase in experimental than control groups in all assessment times
Medeiros [22] (2016)	MPS	23 (23/0) Exp.: 11 Cont.: 12	Exp.: 49.18 ± 11.63 Cont.: 45.83 ± 9.63	Sham-rTMS + IMES	Sham-rTMS + Sham-IMES	VAS Peripheral biomarkers Cortical excitability parameters	End of every session Before After	Significant pain decreases in experimental group than control group There was not any change in all peripheral biomarker’s parameters in both groups
Hadizadeh [23] (2017)	UT MPS	16 (16/0) Exp.: 8 Cont.: 8	Exp.: 24.6 ± 6.4 Cont.: 26.7 ± 6.5	IMES	Sham-IMES	VAS Neck ROM	Before After One week	Significantly higher ROM in IMES group compared to control group one week after treatment No significant differences of pain in the all assessment times
Botelho [24] (2018)	MPS	24 (24/0) Exp.: 12 Cont.: 12	Exp.: 48.36* Cont.: 46*	IMES	Sham-IMES	VAS B-PCP:S Cortical excitability parameters taking analgesic during the treatment	Before After	Experimental group presented lower pain and disability in comparison to control group significantly Analgesic use was 69.4% in sham group and 30.6% in EIMS
Brennan [25] (2020)	MPS	45(37/8) Exp.: 20 Cont.: 25	Exp.: 28 ± 9.99 Cont.: 26.32 ± 8.94	IMES	DN	NPRS NDI	Before 3th week 6th week 12th week	At no time did NDI or NPRS differ significantly between groups

*F* female, *M* male, *n* number, *SD* standard deviation, *Exp*. Experimental, *Cont.* control, *UT* upper trapezius, *MPS* myofascial pain syndrome, *IMES* intramuscular electrical stimulation, *DN* dry needling, *VAS* visual analogue scale, *MPQ* McGill pain questionnaire, *ROM* range of motion, *Flex*. Flexion, *SIMES* sensory intramuscular electrical stimulation, *PPT* pain pressure threshold, *NDI* neck disability index, *rTMS* repetitive transcranial magnetic stimulation, *B-PCP:S* Brazilian profile of chronic pain: screen, *NPRS* numeric pain rating scale

^*^Standard deviation was not reported

Among the included studies, 76 and 82 patients were allocated to IMES and control groups, respectively. Three studies had parallel RCT designs with IMES and control groups [23–25]. Two other studies featured three parallel RCT designs. One compared the effectiveness of low-level laser therapy, IMES, vs. a control group [21]. Another included DN, IMES, and intramuscular stimulation (Gunn-IMS) groups [26]. One study had four groups, including repetitive Transcranial Magnetic Stimulation (rTMS) + IMES, rTMS + sham-IMES, sham- rTMS + IMES, and sham- rTMS + sham-IMES. We considered sham- rTMS + IMES and sham- rTMS + sham-IMES as experimental and control group, respectively in this study [22]. All studies recruited patients with chronic cervical MPS.

In three studies, sham-IMES groups were used as a control group [22–24]. In one study, participants in the control group received prescribed home-based exercises [21], while subjects in another two studies control group received DN [25, 26]. The number of treatment sessions varied from one to ten sessions between studies.

### Risk of bias assessment of selected articles

**Fig. 2:**
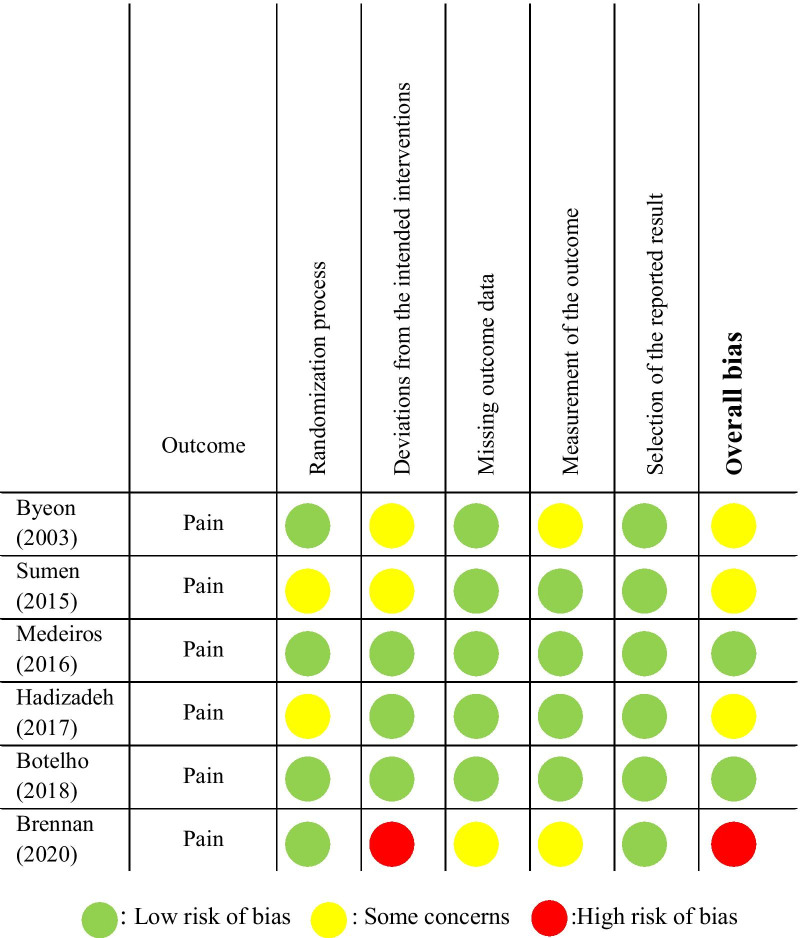
Quality assessment for RCT (RoB 2.0)

Among six included studies, two had low risk of bias [22, 24] and three of them had moderate risk of bias [21, 23, 26]. The study by Brennan et al. [25] was the only study with high risk of bias due to inappropriate intention to treat analysis. Details of the study quality assessment are presented in Fig. 2. The details of the scoring of each item for the included studies are presented in the Additional file 1.

### Wave properties and needle location in IMES group

In four studies, the upper trapezius muscle was treated [21, 23, 25, 26]. Medeiros et al. and Botelho et al. applied IMES to the cervical paraspinal muscles [22, 24]. Only three studies targeted trigger points [21, 23, 25]. The frequencies of the electrical stimulation ranged from 2 to 80 Hz. In two studies, the intensity was increased to the point of contraction [23, 26]. Sumen et al. [21] increased the intensity until the patient sensed the stimulus. Three studies did not report any details about the intensity [22, 24, 25]. Wave properties and IMES technical characteristics are summarized in Table 2.

**Table 2:** The properties of applied intramuscular electrical stimulation of included studies

First author (year)	Location of treatment	Total sessions/session(s) per week/total duration	Wave properties				Needle electrode	Needle in TrP	Reference electrode	
			Shape	Freq. (Hz)	Intensity	Duration of treatment			**Type**	**Pole**
Byeon (2003)	Upper trapezius	6/3/2 w	Biphasic pulse	10	3 times of sensory threshold (contraction level)	15 m	NR	NR	NR	NR
Sumen (2015)	Upper trapezius	10/5/2	Pulse	80	NR (Sensory level)	20 m	NR	Yes	NR	NR
Medeiros (2016)	Dermatomes related to C2-C5 & Paraspinal muscles	10/NR/NR	Pulse	2	NR	20 m	NR	NR	NR	NR
Hadizadeh (2017)	Upper trapezius	1/1/1 d	Biphasic burst	Basic freq.: 120 Burst freq.: 2	NR (Contraction level)	10 m	Cathode	Yes	Patch electrode	Anode
Botelho (2018)	C2-C4 Paraspinal muscle	10/NR/NR	Biphasic pulse	2	NR	20 m	NR	No	NR	NR
Brennan (2020)	Upper trapezius	6/1/6 w	NR	10	NR	10 m	NR	Yes	NR	NR

*TrP* trigger point, *Freq*. frequency, *Hz* hertz, *W* weeks, *m* minute, *NR* not reported, *d* day

### Outcome measures and summary of results

The visual analog scale (VAS) was the most common pain outcome measure. Three studies evaluated ROM measurements [21, 23, 26]. Other outcome measurements included pain by numeric pain rating scale (NPRS), pain pressure thresholds (PPT), biomarkers such as BDNF, pain or functional ability questionnaires, the neck disability index (NDI) and the McGill pain questionnaire (MPQ), and analgesic drug intake (Table 1). Also, the Details of included studies outcome measurements in assessment times (means with standard deviations) are presented in the “Appendix 1: Table 3”.

Byeon et al. [26] compared the effectiveness of IMES and DN; they showed improvement in pain and cervical lateral flexion ROM in all groups, but there were no significant differences of all outcome measurements in all assessment times in both groups [26]. Sumen et al.'s [21] results present statistically significant VAS decreases and PPT increases in the IMES group vs. the control group. Medeiros et al. [22] showed a significant difference in pain reduction between the IMES and control groups but no change in peripheral biomarkers parameters in the experimental and control groups. Hadizadeh et al. [23] showed that ROM was significantly higher in the IMES group than the control group one week after treatment. There were no significant differences in pain in all assessment times between both groups. Botelho et al. [24] showed a significant improvement in pain and analgesic drugs in the IMES group compared to the control group. Brennan et al. [25] compared the effectiveness of IMES and DN; they showed a significant improvement in pain and disability index in both groups and did not NDI or NPRS differ significantly between groups in any assessment times.

### Discussion

The current study is the first systematic review evaluating IMES’s effectiveness in patients with MPS to the best of our knowledge. Six studies with a total of 158 subjects were included in this review. Pain, the most common outcome measurement, was assessed by the VAS and NPRS or the MPQ. The effectiveness of IMES was compared with sham IMES, DN, or no intervention. The number of sessions varied from 1 to 10 sessions. The duration of IMES ranged from 10 to 20 min. The study by Hadizadeh et al. [23] was the only study with a single-session intervention. Three articles reported following the patients from 1 to 6 weeks [21, 23, 25, 26].

In general, studies with a low risk of bias showed a significant improvement in the variables of pain, disability and analgesic use in the IMES group compared to the control group [22, 24]. Also, in studies with moderate risk of bias (Some concerns), reduced pain and improved range of motion have been reported. However, in some cases, there was no significant difference with the control group [21, 23, 26]. In a study with a high risk of bias, no significant difference was reported between the IMES group and the control group in the variables of pain and disability [25].

Initially, we aimed to determine what factors would impact the effectiveness of IMES on MPS, such as the frequency of the applied currents, the duration, the exact location of active and reference needles or electrodes, among others, but the limited number of studies and the heterogenicity among studies did not allow for this kind of analysis. The study by Hadizadeh et al. was the only study demonstrating that one session of IMES could effectively reduce pain and increase ROM not immediately but after a one-week follow-up. It can be due to inflammatory processes after needle insertion, which may present as muscle soreness [27]. How many IMES sessions would be sufficient for clinical improvement cannot be deduced from the current research and requires further study.

There are some mechanisms explaining trigger points. One explanation is offered by the integrated hypothesis, which maintains that trigger points result from repetitive low-intensity trauma, leading to sarcoplasmic retinaculum injury, increased calcium concentration, and permanent contraction in the area. This would result in hypoxia and cell damage in the region [28–30]. It seems that surface, motor excitable electrical stimulation can increase the blood flow; therefore, it can decrease regional hypoxia. Commonly, IMES produces muscle contractions. This method can insert electrical stimulation to the depth of muscle with lower resistance against the current. Therefore, IMES seems to be more effective in managing regional hypoxia in TrP zone compared to superficial ES and the use of DN alone [15, 31]. Besides, most studies used low-frequency current; low frequencies may cause the release of endorphins and enkephalins, leading to a reduction in pain [32].

### Limitations

Our study has several limitations that should be mentioned. First, we included only primary RCT studies in this systematic review, which reduced the number of studies, limiting the ability to generalize the results of this study. Second limitation of this study is that, because the characteristics of the applied electrical stimulation like intensity, pulse duration, frequency, time, and etc. are not fully mentioned in all studies, it is not possible to make recommendations regarding the appropriate parameters. Third, we included RCTs with various type of interventions due to limitation in original studies. Fourth, the small number of included studies and clinical heterogeneity of included studies such as different fallow up point times, different sessions number, different control groups, and outcome measurements did not allow us to pool data and do a meta-analysis on the results. Further research is recommended to do a meta-analysis on this topic after further randomized controlled trials. Fifth, all of the included studies had a small sample size that can impact the result of the ROB2 tool. Therefore, the results of quality assessment in this study should be accepted with this limitation.

Further studies are needed to overcome these limitations. First, more RCT studies with larger sample sizes are needed to compare this intervention with other routine interventions. Second, studies are needed to investigate the placebo effects of this intervention. Studies with objective variables (like TrP size or stiffness found by radiologic methods) are also needed to evaluate this intervention's effectiveness. Also, future studies should include more detailed parameters of the interventions.

## Conclusion

There is preliminary evidence from a few small trials suggesting the efficacy of IMES for the care of myofascial pain syndrome. The data support the conduct of larger trials investigating the efficacy and comparative effectiveness of IMES, and determining the optimal settings and dose of the intervention.

## Supplementary Information

**Additional file 1.** SUP1: The details of the Risk of Bias 2 scoring and rating of each item for the included studies.

## Data Availability

The search strategy, list of excluded studies by title/abstract or full-text screening are available from the corresponding author on reasonable request.

## Appendix

See Tables 3 and 4.

**Table 3:** Details of included studies outcome measurements in assessment times (means with standard deviations)

First author (year)	Outcome measurements		Scale ranges	Treatment groups	Before (Mean ± SD)	Three days (Mean ± SD)	One week (Mean ± SD)	Three weeks (Mean ± SD)	After (Mean ± SD)	Fallow up		
										One week (Mean ± SD)	Four weeks (Mean ± SD)	Twelve weeks (Mean ± SD)
Byeon (2003)	VAS		0–10	Exp	6.2 ± 1.4	5.1 ± 0.9	3.9 ± 1.9		3.1 ± 1.5(2w)			
				Cont	6.2 ± 1.1	5.7 ± 1.5	5.6 ± 1.5		4.5 ± 1.4 (2w)			
	MPQ		0–78	Exp	28.1 ± 15.9	27.3 ± 15.1	24.19 ± 15		22.9 ± 15.5 (2w)			
				Cont	27.6 ± 8	25.3 ± 9	24.6 ± 9.7		20.8 ± 7.3 (2w)			
	ROM		NA	Exp	42.8 ± 3.9	43.6 ± 2.7	44.2 ± 3.3		44.3 ± 2.9 (2w)			
				Cont	42.9 ± 2.6	43.4 ± 2.8	43.9 ± 3.2		43.5 ± 3.1 (2w)			
Sumen (2015)	VAS		0–10	Exp	6.80 ± 1.69				3.40 ± 1.50(2w) *		2.33 ± 1.63*	
				Cont	6.80 ± 1.32				5.00 ± 1.77(2w)		5.20 ± 1.14	
	PPT		NA	Exp	21.80 ± 7.25				29.73 ± 8.85(2w) *		35.93 ± 13.68*	
				Cont	25.30 ± 8.38				26.66 ± 8.37(2w)		25.26 ± 6.54	
	ROM		NA	Exp	32.46 ± 3.71				39.60 ± 5.12(2w)		40.20 ± 4.73	
				Cont	37.00 ± 6.78				41.13 ± 5.12(2w)		40.00 ± 5.42	
	NDI		0–100	Exp	39.33 ± 12.86				26.86 ± 13.33(2w)		21.33 ± 13.65	
				Cont	35.46 ± 9.24				28.40 ± 11.01(2w)		27.73 ± 10.59	
Medeiros (2016)	VAS		0–10	Exp	6.21 ± 2.87				1.95 ± 1.85(10d) *			
				Cont	5.05 ± 2.36				2.38 ± 1.69(10d)			
	PB	BDNF	NA	Exp	19.75 ± 14.06				21.98 ± 11.88(10d)			
				Cont	35.05 ± 42.01				27.78 ± 29.36(10d)			
		S100β	NA	Exp	11.28 ± 4.55				11.87 ± 5.87(10d)			
				Cont	16.68 ± 10.31				18.25 ± 13.72(10d)			
		TNF-α	NA	Exp	28.18 ± 3.34				27.50 ± 16.32(10d)			
				Cont	28.28 ± 7.69				28.72 ± 5.89(10d)			
		IL10	NA	Exp	0.34 ± 1.27				1.17 ± 1.78(10d)			
				Cont	1.11 ± 2.63				1.38 ± 2.47(10d)			
		IL6	NA	Exp	0.48 ± 0.58				0.41 ± 0.44(10d)			
				Cont	0.50 ± 0.60				0.55 ± 0.59(10d)			
		LDH	NA	Exp	89.46 ± 30.38				88.90 ± 44.17(10d)			
				Cont	84.40 ± 46.14				105.22 ± 75.12(10d)			
		GPX	NA	Exp	0.58 ± 0.93				0.55 ± 1.05(10d)			
				Cont	0.40 ± 0.46				0.61 ± 1.53(10d)			
		SOD	NA	Exp	91.55 ± 148.13				85.45 ± 122.21(10d)			
				Cont	50.50 ± 56.75				61.10 ± 95.67(10d)			
		CAT	NA	Exp	716.30 ± 342.93				532.20 ± 580.85(10d)			
				Cont	667.80 ± 549.23				875.70 ± 569.13(10d)			
		CAR	NA	Exp	116.95 ± 34.85				124.30 ± 22.15(10d)			
				Cont	128.55 ± 34.40				121.05 ± 27.60(10d)			
		ROS	NA	Exp	1.02 ± 0.47				0.84 ± 0.82(10d)			
				Cont	0.83 ± 0.78				0.74 ± 0.86(10d)			
	CEP	IF	NA	Exp	1.15 ± 0.29				1.26 ± 0.16(10d)			
				Cont	1.07 ± 0.19				1.09 ± 0.26(10d)			
		SII	NA	Exp	0.28 ± 0.16				0.29 ± 0.09(10d)			
				Cont	0.34 ± 0.14				0.30 ± 0.17(10d)			
		CSP	NA	Exp	74.76 ± 16.82				70.46 ± 21.35(10d)			
				Cont	67.98 ± 20.94				68.69 ± 13.64(10d)			
Hadizadeh (2017)	VAS		0–10	Exp	50.62 ± 10.19				28.62 ± 10.16(IA)	17 ± 13.59		
				Cont	45.25 ± 16.99				33.87 ± 17.54(IA)	35.25 ± 19.57		
	ROM		NA	Exp	28.53 ± 7.31				32.95 ± 8.05(IA)	34.74 ± 3.37*		
				Cont	30.62 ± 7.28				29.24 ± 5.76(IA)	28.72 ± 5.46		
Botelho (2018)	VAS		0–10	Exp	5.53 ± 2.28				2.6 ± 2.4(12 W)*			
				Cont	5.46 ± 2.32				4.01 ± 2.58(12 W)			
	B-PCP:S		0–93	Exp	55.85 ± 14.63				38.11 ± 19.86(12 W) *			
				Cont	56.33 ± 16.23				49.89 ± 14.62(12 W)			
	CEP	MEP	NA	Exp	2.26 ± 0.52				1.84 ± 0.74(12 W)			
				Cont	1.75 ± 0.64				1.69 ± 0.45(12 W)			
Brennan (2018)	NPRS		0–10	Exp	2.95 ± 1.52			2.62 ± 1.59	2.27 ± 1.80(6 W)			1.92 ± 1.63
				Cont	2.59 ± 1.25			2.09 ± 1.07	1.71 ± 1.47(6 W)			1.67 ± 1.45
	NDI		0–100	Exp	0.17 ± 0.10			0.14 ± 0.11	0.11 ± 0.09(6 W)			0.10 ± 0.11
				Cont	0.14 ± 0.09			0.12 ± 0.09	0.09 ± 0.10(6 W)			0.09 ± 0.09

*SD* standard deviation, *VAS* visual analogue scale, *MPQ* McGill pain questionnaire, *ROM* range of motion, *NA* not accessible, *Exp*. Experimental, *Cont*. control, *W* weeks, *PPT* pain pressure threshold, *NDI* neck disability index, *PB* peripheral biomarkers, *BDNF* brain-derived neurotrophic factor, *LDH* lactate dehydrogenase, *GPx* glutathione peroxidase, *SOD* superoxide dismutase, *CAT* catalase activity, *CAR* protein carbonyls, *ROS* reactive oxygen species, *CEP* cortical excitability parameters, *IF* intracortical facilitation, *SII* short intracortical inhibition, *CSP* cortical silent period, *d* days, *IA* immediately after, *B-PCP:S* Brazilian profile of chronic pain: screen, *MEP* motor evoked-potential, *NPRS* numeric pain rating scale

*Statistically significant difference between two groups (P< 0.05)

**Appendix 4:** Excluded studies with justification for exclusion

First author (year)	Title	Reason for exclusion
Stalberg 1987	Intramuscular Stimulation for The Study of Individual Motor End-Plates and Muscle Fibers	Conference paper
Wright 1991	Morphologic and Histochemical Characteristics of Skeletal Muscle After Long-Term Intramuscular Electrical Stimulation	It was not a trial study
Arendt-Nielsen 1998	Assessment Of Muscle Pain in Humans: Clinical and Experimental Aspects	Conference paper
Chu 1999	The Role of The Monopolar Electromyographic Pin in Myofascial Pain Therapy: Automated Twitch-Obtaining Intramuscular Stimulation (ATOIMS) And Electrical Twitch-Obtaining Intramuscular Stimulation (ETOIMS)	Combine IMES with another treatment without control group
Chu 2000	Early Observations in Radiculopathic Pain Control Using Electrodiagnostically Derived New Treatment Techniques: Automated Twitch-Obtaining Intramuscular Stimulation (ATOIMS) And Electrical Twitch-Obtaining Intramuscular Stimulation (ETOIMS)	Combine IMES with another treatment without control group
Gunn 2001	Treating Whiplash-Associated Disorders with Intramuscular Stimulation: A Retrospective Review Of 43 Patients with Long-Term Follow-Up	No electrical stimulation by needle
Karakurum 2001	The ‘Dry-Needle Technique’: Intramuscular Stimulation in Tension Type Headache	No electrical stimulation by needle
Chu 2002	The Efficacy of Automated/Electrical Twitch Obtaining Intramuscular Stimulation (Atoims/Etoims) For Chronic Pain Control: Evaluation with Statistical Process Control Method	Combine IMES with another treatment without control group
Kosek 2003	Perceptual Integration of Intramuscular Electrical Stimulation in The Focal and The Referred Pain Area in Healthy Humans	No electrical stimulation by needle
Chu 2004	Electrical Twitch-Obtaining Intramuscular Stimulation in Lower Back Pain	It was not a randomized control trial
Ga 2007	Intramuscular And Nerve Root Stimulation Vs Lidocaine Injection of Trigger Points in Myofascial Pain Syndrome	No electrical stimulation by needle
Lee 2008	Effects Of Needle Electrical Intramuscular Stimulation on Shoulder and Cervical Myofascial Pain Syndrome and Microcirculation	It was not a randomized control trial
Chu 2008	Etoims Twitch Relief Method in Chronic Refractory Myofascial Pain (CRMP)	No electrical stimulation by needle
Valeriani 2008	Nociceptive Contribution to The Evoked Potentials After Intramuscular Electrical Stimulation	The intervention was not on myofascial pain
Hong-You 2009	Increased H-Reflex Induced by Intramuscular Electrical Stimulation of Latent Myofascial Trigger Point	It was not a trial study
Rainey 2013	The Use of Trigger Point Dry Needling and Intramuscular Electrical Stimulation for A Subject with Chronic Low Back Pain: A Case Report	Combine IMES with another treatment without control group
Jodic 2014	Treatment Of Nonspecific Thoracic Spine Pain with Trigger Point Dry Needling and Intramuscular Electrical Stimulation: A Case Series	It was not a randomized control trial
Borg-Stein 2014	Myofascial Pain Syndrome Treatments	It was not a trial study
Couto 2014	Paraspinal Stimulation Combined with Trigger Point Needling and Needle Rotation for The Treatment of Myofascial Pain: A Randomized Sham-Controlled Clinical Trial	No electrical stimulation by needle
Shin 2014	Intramuscular Stimulation of Peri cranial Myofascial Trigger Points in The Treatment of Frequent Episodic Tension-Type Headache	Conference paper
Fogelman 2015	Efficacy Of Dry Needling for Treatment of Myofascial Pain Syndrome	No electrical stimulation by needle
Chu 2015	Twitch-Obtaining Intramuscular Stimulation: Observations in The Management of Radiculopathic Low Back Pain	The intervention was not on myofascial pain
Calatayud 2016	Improvement Of Myofascial Pain in Equine Brachiocephalicus Muscle Using Dry Needling Technique, A Clinical Commentary	Conference paper
Shanmugam 2016	Effects Of Intramuscular Electrical Stimulation Using Inversely Placed Electrodes on Myofascial Pain Syndrome in Shoulder—A Case Series	It was not a randomized control trial
Ratmansky 2016	Position Statement of The Israeli Society for Musculoskeletal Medicine on Intramuscular Stimulation for Myofascial Pain Syndrome- A Delphi Process	It was not a trial study
Mazloum 2017	Comparative Effects of Dry Needling and Intramuscular Electrical Stimulation with And Without Kinesiology Taping in Patients with Non-Specific Chronic Low Back Pain	Conference paper
Graca-Tarrago 2019	Intramuscular Electrical Stimulus Potentiates Motor Cortex Modulation Effects on Pain and Descending Inhibitory Systems in Knee Osteoarthritis: A Randomized, Factorial, Sham-Controlled Study	The intervention was not on myofascial pain
Moon 2019	Intramuscular Stimulation as A Novel Alternative Method of Pain Management After Thoracic Surgery	The intervention was not on myofascial pain
Kim 2019	A New Treatment Modality for The Postoperative Muscular Pain Management in Pylorus Preserving Pancreaticoduodenectomy: A Double-Blind Randomized Control Trial	Conference paper

## Abbreviations

MPS: Myofascial pain syndrome
IMES: Intramuscular electrical stimulation
RCTs: Randomized controlled trials
RoB2: Revised cochrane risk-of-bias tool for randomized trials
TrP: Trigger point
ROM: Range of motion
DN: Dry needling
ES: Electrical stimulation
MeSH: Medical subject heading
rTMS: Repetitive transcranial magnetic stimulation
VAS: Visual analog scale
NPRS: Numeric pain rating scale
PPT: Pain pressure thresholds
NDI: Neck disability index
MPQ: Mcgill pain questionnaire
