# Supplementary material for: The efficacy of intramuscular electrical stimulation in the management of patients with myofascial pain syndrome: a systematic review
Source: Chiropr Man Therap. 2021 Sep 27;29:40. doi: 10.1186/s12998-021-00396-z (PMC8477566; doi:10.1186/s12998-021-00396-z)
Supplement: Supplementary file 1 — Additional file 1. SUP1: The details of the Risk of Bias 2 scoring and rating of each item for the included studies. [file 12998_2021_396_MOESM1_ESM.docx]

| SUP1: The details of the Risk of Bias 2 scoring and rating of each item for the included studies. | | | | | | | | | | | | | | | | | | | | | | | | | | | | |
| --- | --- | --- | --- | --- | --- | --- | --- | --- | --- | --- | --- | --- | --- | --- | --- | --- | --- | --- | --- | --- | --- | --- | --- | --- | --- | --- | --- | --- |
| First Author (year) | D1: Randomization process | | | | D2: Deviations from the intended interventions | | | | | | | | D3: Missing outcome data | | | | | D4: Measurement of the outcome | | | | | | D5: Selection of the reported result | | | | Overall bias |
|  | 1.1 | 1.2 | 1.3 | ROB | 2.1 | 2.2 | 2.3 | 2.4 | 2.5 | 2.6 | 2.7 | ROB | 3.1 | 3.2 | 3.3 | 3.4 | ROB | 4.1 | 4.2 | 4.3 | 4.4 | 4.5 | ROB | 5.1 | 5.2 | 5.3 | ROB |  |
| Byeon  (2003) | NI | NI | N |  | NI | NI | N | - | - | NI | PN |  | Y | - | - | - |  | N | N | NI | NI | PN |  | Y | N | N |  |  |
| Sumen  (2015) | Y | NI | N |  | N | Y | NI | - | - | PY | - |  | Y | - | - | - |  | N | N | N | - | - |  | Y | N | N |  |  |
| Medeiros (2016) | Y | Y | N |  | N | - | - | - | - | Y | - |  | Y | - | - | - |  | N | N | N | - | - |  | Y | N | N |  |  |
| Hadizadeh (2017) | Y | NI | N |  | NI | N | N | - | - | Y | - |  | Y | - | - | - |  | N | N | N | - | - |  | Y | N | N |  |  |
| Botelho  (2018) | Y | Y | N |  | NI | N | N | - | - | Y | - |  | Y | - | - | - |  | N | N | N | - | - |  | Y | N | N |  |  |
| Brennan  (2020) | Y | NI | N |  | PY | Y | N | - | - | PN | PY |  | PN | PN | NI | PN |  | N | N | Y | PY | PN |  | Y | N | N |  |  |
| D: Domain; ROM: Risk of Bias; NI: No Information; N: No; PN: Probably No; Y: Yes; PY: Probably Yes.   \|  \| Low risk of bias \|  \| Some concerns \|  \| High risk of bias \| \| --- \| --- \| --- \| --- \| --- \| --- \| | | | | | | | | | | | | | | | | | | | | | | | | | | | | |
